# HPB News

**DOI:** 10.1155/1990/16427

**Published:** 1990


					HPB Surgery 1990, Vol.2, p.149
Reprints available directly from the publisher
Photocopying permitted by license only
1990 Harwood Academic Publishers GmbH
Printed in the United Kingdom
HPB NEWS
HPB IN SOVIET UNION
Ministry of Health of the USSR, All-Union Scientific Society of Surgeons, decided
at a meeting on November 28, 1989, to set up a hepato-pancreato-biliary surgery
section of All-Union Society of Surgeons. Professor Eduard Galperin was
appointed chairman of the section. It was decided that the section should partici-
pate in the activities of the World HPB Surgery Association.
After this the HPB World Association has received 25 new members from Soviet
Union. The number of members in the Soviet Union is at present 27.
The national delegate for Soviet Union is
Professor Eduard I. Galperin
Department of Surgery
1st Moscow Medical Institute
Hospital 7
Kolomensky Proezd 4
MOSCOW
Soviet Union
HPB WORLD ASSOCIATION
Some countries have appointed new national delegates to the HPB World
Association.
Country:
Denmark
Delegate:
Susanne Lone Jensen, M.D.
Surgical Gastroenterological
epartment L
rhus Kommunehospital
DK-8000/RHUS C
New Zealand Richard Stubbs, M.D.
Department of Surgery
Wellington School of Medicine
Wellington Hospital
P.O. Box 7343
Wellington South
149

				

